# Ulnar and Superficial Radial Nerve Swellings in Two Patients with Leprosy

**DOI:** 10.4269/ajtmh.17-0940

**Published:** 2018-04

**Authors:** Neeraj Kumar, Ravindra Kumar Garg, Hardeep Singh Malhotra

**Affiliations:** Department of Neurology, King George Medical University, Lucknow, Uttar Pradesh, India

**Figure 1.**
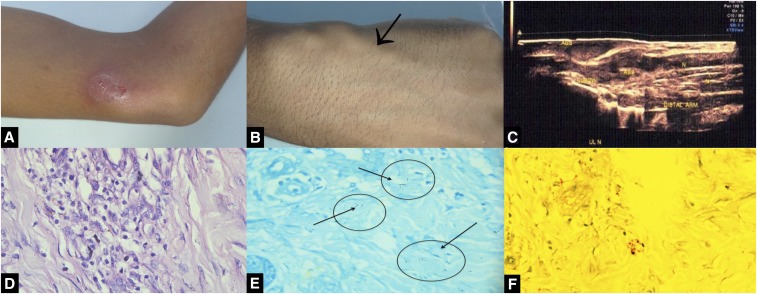
Image showing swelling and signs of inflammation at the ulnar aspect of the lower arm region (**A**), enlargement of the left superficial radial nerve (**B**), ultrasound depiction of subcutaneous and ulnar nerve abscesses (**C**), higher magnification showing inflammatory infiltrate containing foamy histiocytes and lymphocytes (H and E; ×400) (**D**), modified Ziehl–Neelsen–stained section showing acid-fast bacilli (**E**), and modified Ziehl–Neelsen–stained section showing acid-fast–stained lepra bacilli (red dots and rods) (**F**). This figure appears in color at www.ajtmh.org.

**Figure 2.**
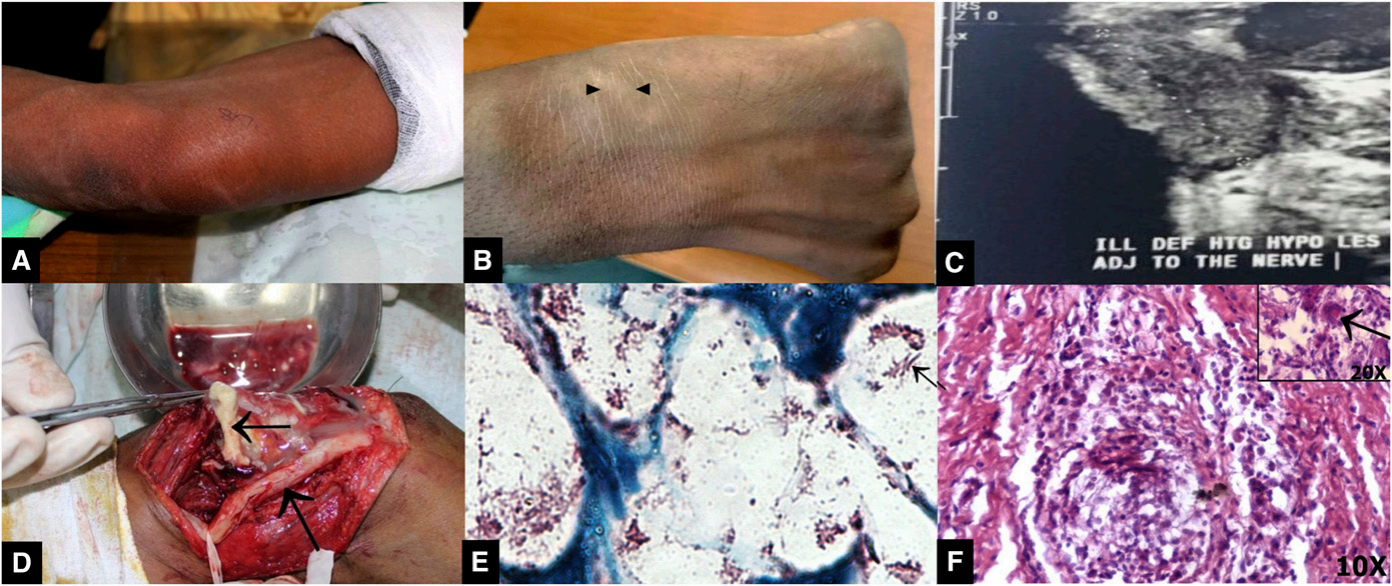
Image showing swelling and signs of inflammation at the ulnar aspect of the lower arm region (**A**), enlargement of the left superficial radial nerve (**B**), ultrasound depiction of subcutaneous and ulnar nerve abscesses (**C**), operative image showing the thickened ulnar nerve and pus drained from swelling (**D**), acid-fast bacilli (**E**), and Langhans giant cells (**F**). This figure appears in color at www.ajtmh.org.

Localized nerve swellings are frequently caused by neurofibroma, plexiform neurofibroma, malignant peripheral nerve sheath tumor, and neural lipoma. Many non-tumor causes, such as intraneuronal ganglion cyst, pseudoneuroma, and leprosy, can also produce localized nerve swellings.^1^ Here, we describe two patients who presented with painful localized nerve swellings. Nerve swellings were present along the course of the ulnar nerve and superficial radial nerve, in both patients, and wasting of hand muscles along with associated sensory loss was present. No skin lesion was seen. A nerve conduction study revealed ulnar mononeuropathy, in both cases. Slit smear examinations were negative. An ultrasound evaluation showed ulnar nerve abscesses. Surgery was performed to drain the abscess. Histopathological evaluation of necrotic biopsied material, in both cases, showed granuloma and an acid-fast bacillus stain demonstrated *Mycobacterium leprae* (Figures 1 and 2). Patients were given multidrug therapy for 12 months. After 3 months, sensory and motor functions were restored.

Nerve abscesses usually present with single or multiple reddish, tender fluctuant swellings on the medial aspect of the forearm, along the course of the nerve. Nerve abscesses may paradoxically develop even after successful multidrug therapy.^2^ An enhanced immune reaction results in liquefaction of caseous nerve lesions leading to abscess formation.^3^ Nerve abscesses are more common in borderline and lepromatous leprosy.^4^ In lepromatous leprosy, abscesses may be due to an erythema nodosum leprosum. In tuberculoid type of leprosy, type 1 reversal reaction, after multibacillary multidrug therapy, results in an immune reconstitution inflammatory syndrome with formation of nerve abscesses.

Ultrasonographic studies can efficiently demonstrate nerve abscesses in leprosy. Ultrasound evaluation reveals a hypoechoic lesion with fascicular pattern disorganization and a round anechoic area. Magnetic resonance imaging reveals a nerve abscess as hypointense lesion on T1-weighted images and hyperintense on T2-weighted images along with peripheral rim enhancement with central necrosis.^5^ Fine-needle aspirates from the abscess may reveal elements of granuloma with lepra bacilli, thus helping in diagnosis. Leprosy nerve abscesses may be misdiagnosed as tuberculous lymphadenitis, schwannoma, lipoma, tuberculoma, and bacterial nerve abscess. These patients respond well to surgery and multidrug therapy.^2^
